# Supplementary material for: Are carbon emissions trading and green financial instruments synergistic? -Comprehensive quantitative research based on content analysis
Source: PLoS One. 2024 Mar 7;19(3):e0298601. doi: 10.1371/journal.pone.0298601 (PMC10919601; doi:10.1371/journal.pone.0298601)
Supplement: S1 Data — (ZIP) [file pone.0298601.s001.zip › Data/地级市Z_F工作报告.zip!/╣π╢1⁄2/╜╥╤⌠╩╨/└┤╫╘╣1⁄2╓┌║┼í╛┬φ┐╦╩2╛▌═°í┐_2015.docx]

各位代表：

现在，我代表市人民政府向大会作工作报告，请予审议，并请市政协各位委员和其他列席人员提出意见。

2014年工作回顾

过去一年，在省委省政府和市委的正确领导下，在市人大及其常委会和市政协的监督支持下，我们紧紧围绕“打造粤东发展极，建设幸福新揭阳”的核心任务，牢牢把握“强化效率、彰显公平、惠民执政、绿色发展”的施政要求，主动适应经济发展新常态，积极应对各种困难挑战，全力推动经济社会发展，较好地完成了市五届人大四次会议确定的各项目标任务。

（一）经济发展稳健提质。2014年，全市生产总值1780.44亿元，比上年（下同）增长10.7%；规模以上工业增加值1047.18亿元，增长15.8%；固定资产投资1093.8亿元，增长39.5%；社会消费品零售总额759.02亿元，增长15.4%；外贸进出口54.6亿美元，增长16.4%；地方公共财政预算收入73.69亿元，增长10.5%。金融机构各项存款余额1709.93亿元，增长11.8%；贷款余额884.94亿元，增长21.4%；存贷比51.75%，比年初提高4.09个百分点。工业用电量117.5亿千瓦时，增长15%。

（二）“三大建设”扎实推进。揭阳航空口岸通过国家验收，揭阳潮汕机场成为全省3个国际机场之一。汕湛、潮惠、揭惠3条高速公路揭阳段累计完成投资71.96亿元。梅汕高铁揭阳段、疏港铁路、广梅汕铁路改线工程前期工作顺利。改造国省道69.4公里、地方公路504公里。空港经济区、海港经济区完成投资237.44亿元，中委石化炼油、中海油粤东LNG一体化等项目加快推进，佳龙炼化一体化项目正式签约。中德金属生态城“零排放”表面处理中心、“四大中心”、进园道路基本建成；海峡两岸食品药品合作区、纺织产业生态园等园区规划建设取得新进展。改革城乡规划决策管理机制，编制《揭阳市中心城区扩容提质建设规划》等规划，蓝城区与产业转移工业园实现一体化发展。发行城投债16亿元，争取省粤东西北股权基金9亿元，揭阳新区一批市政项目启动建设，空港新城、揭东新城、玉都新城拉开框架。“三旧”改造步伐得以加快。

（三）产业建设形成热潮。产业强市理念深入人心，实施“产业引进”和“产业登高”工程，每年安排50%以上土地指标用于工业项目。与阿里巴巴集团、京东公司等开展战略合作；完成工业投资572.2亿元，其中，工业技改投资126.81亿元。被列为省金融科技产业融合创新综合试验区，引进6家金融机构，5家农信社全部改制为农村商业银行，设立5个普惠金融服务中心，1家企业“新三板”挂牌，13家企业成功发行集合绩优债；新设院士工作站2个；石化、纺织服装、医药、金属、食品五大产业完成增加值680.21亿元，增长17.2%；玩具产业出口增长51.1%。电商8610计划全面铺开，国家电子商务示范城市、工业电子商务创新发展示范城市建设扎实推进，军埔等9个村入选“中国淘宝村”，普宁占陇镇成为首批19个“中国淘宝镇”之一。全市快递业务量增长259%，增速列全省第1位。

（四）国际合作拓开局面。实施与德国为重点的国际合作，主动承接德国产业溢出，中德资源再生基地等3个项目参加国家高访活动签约，承办中德两党第二次可持续发展对话会等高端活动，中德金属生态城成为中德经济合作委员会成员，被定为省级国际科技合作基地。市金属企业联合会在德国设立6个办事处，与德国工商大会、德国雇主协会等9家协会建立联盟关系，与埃斯林根应用科技大学等20多家德国科研机构和中介机构建立合作关系。承办中联部非洲7国青年电商领袖培训班，与荷兰、法国、葡萄牙和缅甸等国家的港口、服装、鞋业、玉器等产业合作扎实推进。

（五）“三农”工作提速增效。与省农业厅共建农业“一镇一品”工程示范市，建成30个“一镇一品”示范镇，培育30个农业品牌产品，示范镇农业总产值增长8%。引进省供销社建设粤东农产品物流城。2014年，农林牧渔业增加值162.37亿元，增长4.3%，增速全省第一。投入水利建设资金8.46亿元，建成普宁白坑湖水库综合整治工程等一批重点水利工程。扶贫开发“双到”共落实帮扶资金7.4亿元，扶持贫困村项目3208个，扶持贫困户项目9.75万个。实施“乡贤乡村建设”、“农村清洁工程”，建成69座镇生活垃圾转运站和3410个自然村垃圾收集点。完成村级“两委”换届选举工作。

（六）民生事业协同发展。年度财政民生投入148.8亿元，占预算支出79.4%。投入教育经费62亿元，建设义务教育标准化学校372所，新（改、扩）建幼儿园96所；40个镇通过省教育强镇验收，揭东区成功创建省教育强区并通过“全国义务教育发展基本均衡区”认定。高考取得历史性突破，上省线37887人。举办市第六届运动会暨第三届中学生运动会。医改和计生工作得到加强，医保参保率达到98%，市人民医院、慈云医院等重点项目建设进展顺利，康美医院正式运营，与中大、南方医科大及附属医院合作取得显著成效。2014年十件民生实事基本落实，百岁老人高龄津贴提高至每月400元；继续为本科以上贫困大学生每月发放200元生活补助；给全市乡镇卫生院全日制本科学历以上医生每月发放500元人才津贴；开工建设保障性住房2963套，改造农村困难户危房5500户；在揭西创立农村留守老人、儿童关爱基金，建设留守老人关爱中心、留守儿童学校、儿童友好社区示范点和农村应急避险中心；组建10支抢险轻舟队，完善基层救灾应急队伍；危桥改造项目完成工程量90%；粤东残疾人集中就业培训基地启动建设；揭阳一中科技及文化中心在规划之中。市社会福利中心基本完成。在全省率先推出政府民生综合责任保险。“一村（社区）一法律顾问”工作走在全省前列，信访维稳工作不断强化，社会治安保持稳定。安全生产和消防安全形势持续好转，实现食品药品安全“零事故”。

（七）环保生态得到加强。电镀产业基地完成主体建设；纺织服装漂染园、金属酸洗产业园启动前期工作；完成省污染减排计划项目68宗；淘汰印染落后产能5430万米，单位GDP能耗下降3%；榕江、练江污染整治力度不断加大，大气污染整治初见成效。启动美丽海湾建设，海洋生态环境有效改善。开展城市绿化大行动，完成碳汇造林14.6万亩、生态景观林带119公里，扩大生态公益林面积20.45万亩，新增森林公园12个、湿地公园1个，建设乡村绿化美化示范村322个，全市森林面积438.56万亩，森林覆盖率达到55.26%。

（八）体制改革不断深化。完成新一轮政府机构改革，开展部门权责清单编制试点，理顺城市管理体制。推进商事登记和行政审批制度改革，取消、下放、转移市级审批事项50项，审批“前置改后置”108项，新登记企业和注册资本分别增长53.2%和171.3%。成立市人民政府行政复议委员会，制定和修订市政府规范性文件8件。办理市人大代表建议、市政协提案和建议意见154件。深入开展党的群众路线教育实践活动，坚决反对“四风”，市政府系统议事协调机构撤销49%，全市财政“三公”经费压缩13.4%。严格执行中央“八项规定”、国务院“约法三章”，全面落实政府系统党风廉政建设责任制，强化行政监察和审计监督，政府自身建设得到有效加强。

此外，在全省率先开展阳光征兵试点。统计、气象、地震、物价、旅游、文化、档案、史志、海洋渔业、妇女儿童、人民防空、民族宗教、侨务和港澳台、红十字、社科、文联等工作也取得了新进步。

各位代表！过去的一年，我们实现的进步，离不开省委省政府和市委的正确领导，离不开广大乡贤侨胞、各界人士和国际友人的关心支持，是全市干部群众团结拼搏、奋发进取的结果。在此，我代表市人民政府，向全市广大干部群众致以崇高敬意！向关心支持揭阳改革发展的各界朋友、港澳台同胞、海外侨胞和国际友人表示衷心感谢！

同时，我们也清醒地认识到，当前我市还存在不少困难和问题：思想观念与新常态要求不相适应；创新驱动发展能力不足；经济转型依然任重道远；环保治污任务繁重；民生事业欠账较多；社会管理压力较大，等等。对此，我们将高度重视，在加快发展中逐步解决。

2015年工作安排

2015年是全面深化改革和推进依法治国的关键之年，也是全面完成“十二五”规划目标任务的决胜之年。当前，我国经济发展进入新常态，认识新常态，适应新常态，引领新常态，是当前和今后一个时期地方经济发展的大逻辑。我们要遵循经济规律、自然规律和社会规律，科学认识当前形势，准确判断未来走势，顺势而为，乘势而上，在新的历史起点上开创经济社会发展新局面。

今年政府工作总的要求是：深入贯彻落实十八届三中、四中全会和省委十一届四次全会、市委五届七次全会精神，紧紧围绕“打造粤东发展极，建设幸福新揭阳”的核心任务，主动适应经济发展新常态，坚定产业强市理念，狠抓改革攻坚，突出创新驱动，强化两大经济引擎，全力推进三大建设，不断拓展国际合作，大力发展实体经济，切实增进民生福祉，着力推进依法行政，全面推动揭阳在加快发展中转型升级。

今年主要预期目标是：生产总值增长12%左右；规模以上工业增加值增长17%；固定资产投资增长35%；社会消费品零售总额增长16%；外贸进出口增长13%；地方公共财政预算收入增长13%；居民消费价格涨幅控制在3%左右；居民收入增长与经济增长基本同步；城镇登记失业率控制在3.5%以内；人口自然增长率控制在8‰以内；节能减排约束性指标完成省下达年度计划。

为实现上述目标，重点抓好以下工作：

（一）增强“两港经济”新动力

增创空港经济竞争优势。支持揭阳机场、南航揭阳基地建设及进港航空公司拓展市场，争取客流、物流有重大突破。支持中外运100万标箱榕江港的整合建设，创建物流聚集区。实施空港传统产业“登高工程”，集中力量推进模具、石材产业的组织化建设和技术改造、研发、交易平台建设，形成产业链。依托潮汕玩具产业的沉淀积累，着力创建玩具研发设计、电子商务、仓储物流、检测中心、交易平台及人力资源培训服务等生产性服务业，建设潮汕玩具产业高地。加快空港新城建设，建设有产业依托、交通支撑的绿色生态新城。

拓展海港经济发展格局。把握海洋经济发展规律，彰显区域优势资源，科学规划，精深开发。以21世纪海上丝绸之路重要节点作定位，规划建设连接海西与珠三角、面向全球的南方重要产业港口，发展临港产业物流园区。加快推进中委炼油项目建设，完善大南海石化工业区基础设施，争取承接国家“重大工程包”；着眼国内外知名石化企业，开展产业链招商；创建民营石化产业转型升级园区，实施潮汕石化企业集群回归工程，建设南方石化产业基地。以中海油粤东LNG一体化项目、中电投综合项目等为带动，创建临港工业经济带。着眼港口经济区发展，对惠来城区布局作再谋划，以港促产、以产促城，形成港产城互为依托、协同发展新格局。

（二）推动“三大建设”新突破

构建立体交通新体系。加快汕湛、潮惠、揭惠等高速公路揭阳段建设，实现县县通高速目标；开工建设揭惠高速市区连接线，改造提升206国道等一批国省道和地方公路，抓好干线公路“迎国检”项目建设；推进梅汕高铁揭阳段、广梅汕铁路改线、疏港铁路规划建设；实施榕江航道新一轮整治工程，联合中外运培育榕江航运物流带；推进与荷兰鹿特丹港务局合作，建设空港、海港、河港、高铁、高速五位一体，客流、物流双流汇合的区域交通枢纽型平台。试点开辟新能源公交系统，建设综合客货运输基地。投资6.8亿元建设通信基础设施，推进宽带提速普及，扩大物联网、大数据等信息技术应用，让揭阳成为互联网时代的“云端”城市。

打造特色产业平台。以园区建设为切入，坚定县域产业主题，创建县域产业平台，形成县域全产业链，扎实建设“10+2”产业集群。榕城区着力街区建设，培育发展后汽车服务业、古城文化旅游业，建设鞋业创意文化区。揭东区以中德金属生态城为主抓手，建设绿色电镀产业聚集区、金属制造业聚集区、中德中小企业合作区和中德跨境电子商务产业园；以军埔电商村的溢出，建设电商产业聚集区，实现先进制造业与现代贸易业双轮并驱。蓝城区以玉产业转型升级启动文化创意产业的构建，以海峡食品药品合作区、国家级快递物流产业园区及电子商务中小微加工业聚集区建设，实现增量的培育。空港经济区强化与机场、航空公司的全面深度合作，以空港优势吸引物流产业聚集，以物流优势形成产业培育优势，当前重点助推模具、石材产业转型升级，助推玩具产业创建出口基地。普宁加快中法纺织服装生态城规划建设，以绿色环保要素，重铸纺织产业竞争力；以资本要素引导人才、科技等创新要素聚集，创建医药产业高地。揭西继续对大北山旅游区深耕细作，创建健康生态旅游产业带；引导乡贤企业集群回归，建设电线电缆转型升级产业区。惠来坚持滨海制造业、滨海物流业、滨海旅游业并举，形成临海产业大格局。大南海石化工业区在石化产业链中把握关键环节，实现产业链连接延伸。普侨区、大南山侨区坚持与普宁及惠来、大南海主动对接，重点发展生产性服务业、健康养老产业、绿色生态农业及农产品加工配送业。

建设产城融合活力之城。完善城乡规划体系，强化规划引领作用。推进新区建设和旧城改造，优化经济发展空间布局，引领可持续城镇化建设。新区坚持产城融合理念，以产带城加快开发建设步伐；建设一批道路、公园等市政项目，配置好教育、医疗、文化、体育等公共资源；严格执行容积率指标，保障公共空间和绿地水域面积。旧城突出延续历史文脉，抓好棚户区和“三旧”改造，强化公共服务配套；下大力气，集中资源，培育街区经济。争取国内知名开发企业进驻揭阳，提高城市品位。推进各县城和重点镇建设，加快城镇化进程。融入汕潮揭同城化，在粤东城市群崛起中发挥积极作用。

（三）增创产业发展新优势

建设实体经济强市。依托国家“一带一路”战略，深化对德合作，积极对接德国“工业4.0”，实现后发地区的先发优势。加快中德金属生态城建设，完善基础设施配套，创建德国产业溢出承接平台，加快中德合作落地项目竣工达产，形成项目的“灯塔”示范效应，建设中德中小企业合作聚集区；举办中德中小企业合作交流会，围绕“4个100”的目标（100家中德中小企业对接、交流、合作；100名德国专家工程师签约进厂入企；100项高新科技成果和先进设备展示、转让、转化；创立100亿中德中小企业合作基金）创建中德中小企业合作交流平台，以平台招商、集群招商形成对德中小企业的成体系引进；创立中德合作科技孵化器，强化科技支撑；引入阿德勒斯霍夫科技园区的管理经验，创新中德金属生态城体制机制。以中德金属生态城为示范，推进揭阳产业的全面国际合作，实现产业要素的引进、集聚、合成，形成产业活力、竞争力。

深入实施产业引进。全面实施产业引进工程，围绕产业主题，依托产业平台，结合产业链条，确定引进目标，列出引进清单，制定引进计划，落实引进责任，开展引进竞赛，兑现引进奖励。强化项目落地服务，从土地、金融、人力资源、政务服务等方面予以全方位、高效率保障，营造“零障碍”项目建设环境。着力产业链引进，力争引进一个龙头项目带进一批上下游项目，形成“1+N”产业引进效应。

继续推进产业登高。以创新驱动为总抓手，以6166计划为工作体系，以技术改造为工作切入，继续狠抓产业登高。有计划有针对性地举办企业家培训班，强化产业人才队伍建设；扎实推动产业组织化建设，强化行业协会作为，重点支持行业协会创建产业公共产品，形成公共服务体系；组织科技、金融、人才资源支持产业技术改造，统筹推进技术创新、产品创新、组织创新、商业模式创新、市场创新、产业合作创新，以扎扎实实的举措、资源培育100家产业技改示范企业。在中德金属生态城建立金融科技产业融合创新试验区，创新发展产业金融、科技金融、国际金融和普惠金融，大力推动企业上市融资，集合金融资源破解企业融资难问题。推进征信体系建设，营造良好的产业生态。实施质量强市战略和技术标准战略，建立产品质量检测公共服务平台，积极创建省技术转移集聚区和知识产权保护试验区。制订出台加快我市现代服务业发展的政策措施，规划建设区域总部经济基地，大力发展现代物流、科技服务、工业设计、文化创意等生产性服务业，大力发展旅游业，促进服务业发展提速、比重提高、水平提升。

大力培育电子商务。围绕建设21世纪海上丝绸之路电商港的目标，与阿里、京东等开展深度合作，共建电商产业先行市。依托电商培训大联盟，推进十万电商人才大培训，建设电商人才资源强市。加快打造六大电商集聚区，推动六大优势产业集群上网，建设网上揭货高地。创建中德跨境电子商务产业园，建设中德跨境电商平台和海关特殊监管区。以农业“一镇一品”为切入，实施电商下乡工程，在揭东、普宁、揭西、惠来铺开县级服务中心和镇村服务站建设。出台快递物流仓储专项规划和扶持政策，创建国家级快递物流试验区，推动菜鸟网络和京东仓储物流项目落户建设。联合北外创立电商创新学院，推进电商征信体系建设，出台电商管理条例，建设电商文化体系，形成良好的电商发展生态。

探索发展现代职教。推进与德国埃斯林根应用科技大学合办中德双元应用科技大学，联合德国国际合作机构、巴伐利亚州经济教育集团等机构试行开展“双元制”职业培训项目。支持揭阳职业技术学院按照“区域化、市场化、开放化、品牌化”要求，提升办学质量。在大南海石化工业区引进北京化工大学设立职业教育院校；在蓝城区创建揭阳卫生职业技术学院；支持在普侨区建设医药健康职业技术学院；探索在普宁市建设职业教育示范区。组建现代职业教育联盟，推动每所职业院校对接1个以上行业协会，探索产教融合的现代职业教育新路子，打造产业人才高地。

（四）促进“三农”工作新提升

做强“一镇一品”工程。围绕结对一个农业科技院所、确定一个拳头产品、培育一个龙头企业、扶持一个专业合作社、建设一个示范基地、打造一种营销模式、形成一个农业品牌、实施一项农产品上网工程“八个一”，推动“一镇一品”提质做强。实施电子商务下乡和“一镇一品”上网工程，打造军埔为旗舰、十个先导镇为引领的“1+10”农产品电商示范体系，创建一批省市共建及市、县、镇自建的电商农产品供应示范基地。

加强农村基础建设。加快榕江大围达标加固、惠来中东部供水等重点水利工程建设，提高防灾减灾能力。做好引韩供水工程前期工作，推进村村通自来水工程。开展永久性基本农田划定工作，完成13.73万亩高标准农田建设，抓好粮仓改造建设和粮储工作，确保粮食安全。按“户收集、村集中、镇转运、县处理”的要求深入开展农村卫生整治，推进实施“乡贤乡村建设”工程，美化乡村环境。加大资源政策倾斜，深入开展扶贫开发“双到”工作，年度实现脱贫6.3万人。

增强农村发展活力。有序推进农村土地承包经营权确权登记颁证工作，稳妥做好农村集体土地征收、宅基地制度等改革试点。探索开展农村集体资产股权股份制度改革试点，推动农村集体“三资”管理服务平台全覆盖。积极引入农业现代经营，鼓励发展农业综合经营，着力推进与广东农垦集团、省供销社共建农产品物流园和粤东农产品物流城。

（五）力促社会事业新进步

全面实现教育创强。组建教育发展基金，搭建教育融资平台，加大教育投入力度。推进与北外、北师大、华师大等高校合作，着力打造揭阳一中等一批名校。改造提升8所薄弱普通高中，推进义务教育均衡优质标准化发展，力争义务教育标准化学校覆盖率达到100%，实现创建教育强市目标。整合盘活存量校舍资源，统筹发展学前教育、老年教育和特殊教育。推进“强师工程”，着力打造一支有理想信念、道德情操、扎实学识、仁爱之心的教师队伍。

提升城市文化内涵。强化文化载体建设，完善市博物馆、群艺馆及大剧院配套建设，向公众开放文化设施；实施榕城古城保护开发工程，启动丁日昌旧居、骑楼街区修缮工程；依托揭阳学宫创办国学讲堂，弘扬优秀传统文化。创立乡土文化基金，培育乡土文化人才，扶持乡土文化社团，推动文化下农村、进社区。举办中德文化艺术节，推动潮汕文化与日耳曼文化互鉴发展。大力宣传倡导社会主义核心价值观，培育提升市民素质。塑造独树一帜的产业文化。支持发展社工和志愿者队伍，打造城市流动的风景线。

完善健康服务体系。深化医药卫生体制改革，重点推进揭西县级公立医院综合改革试点，扩大平价医院、平价诊室、平价药包和基本药物制度范围。加快市人民医院门急诊住院楼、慈云医院扩建工程和揭阳中医院、普宁中医院、惠来人民医院等龙头医院建设，推进乡镇卫生院“五个一”工程，鼓励支持社会办医，优化我市医疗资源配置。深化与省人民医院、中大医学院等合作，推进医疗信息化建设，实现市县医疗资源互通共享。引进社会化养老服务机构，大力发展健康服务业。加强计划生育服务，稳定低生育率水平。

推进其他事业发展。按省要求提高养老、低保、五保、孤儿、残疾等保障标准，推进社保扩面征缴。推进全民健身战略，大力发展体育产业。研究编制“十三五”规划和各项专项规划。启动创建国家一级档案馆工作。做好新闻出版、广播电视、气象、地震、国防动员、人民防空、双拥、民族宗教、红十字、社科、史志、文联等工作。

（六）办好惠民利民新实事

坚守底线，突出重点，加快补齐短板，更加务实办好十件民生实事。一是为全市人民统一购买“政府民生综合责任险”，为全市70岁以上老人投保意外伤害险。二是完善高龄津贴制度，百岁老人每人每月500元，80-99周岁老人津贴标准由各县（市、区）根据实际制订。三是首期投入100辆新能源汽车，65周岁以上老人和残疾人免费乘坐市区环城公交。四是免除公立幼儿园在园低保户儿童保教费。五是实施电商下乡工程，创建千家农村电商服务站。六是建设千个“快递E站”和万个快递智能箱。七是在市区新建24座公厕，新设置3140个果皮箱。八是新增划定1000个市区免费公共临时停车位。九是开工建设保障性住房、棚户区改造住房共11286套（户）。十是建设11座列入国家和省责任书项目的污水处理厂。

（七）构筑生态文明新景象

强力整治环境污染。认真贯彻执行新《环保法》，以壮士断腕的决心、背水一战的气概，向污染宣战。按属地管理原则落实“河长制”，推进练江、榕江流域水环境综合整治。将2015年确定为全市污水处理设施建设年，采取PPP等方式建设市区污水处理厂二期和玉滘、占陇等11座列入国家和省责任书项目的污水处理厂及相关配套管网，切实提高工程减排能力。建成运行“零排放”电镀园区，狠抓重金属污染整治，加快启动酸洗、漂染行业污染治理，同步启动食品药品合作区治污工程。强化大气污染联防联控，深入整治PM10、PM2.5和臭氧等污染，提升空气污染治理成效。推动环境行政执法与刑事司法相衔接，依法从严查处环境违法行为，让违法者付出高昂代价。

推进森林进城围城。深入贯彻“人树一体”理念，以森林碳汇、生态景观林带、森林进城围城、乡村绿化美化四大工程建设为重点，推进新一轮绿化揭阳大行动，让森林进社区，让大树入门庭，让公园进新区，努力创建国家园林城市。抓好中德万亩森林公园、黄岐山森林公园、紫峰山森林公园、奥林匹克生态公园等建设，新增造林12万亩，新增县镇两级森林公园12个，森林覆盖率提高至55.32%。

坚定守卫城市资源。坚持“亩产论英雄”，建立用地效益动态评估制度，加大“三旧”改造力度，促进节约集约用地。严守耕地红线，坚决遏制违法违规用地行为。加强岸线使用审批和清理，整合利用岸线港池资源，开展海岸带综合整治修复，推进美丽港湾建设。

（八）形成依法治市新风尚

强化依法治理。以惠来毒品问题整治为重点，在全市范围内掀起一场禁毒人民战争，重拳打击制毒、贩毒、吸毒行为，建设特殊吸毒群体收治场所，坚决铲除毒品犯罪土壤。扎实推进公安“四项建设”，深入开展“3+2”专项整治行动，严厉打击各类违法犯罪，维护社会平安稳定。落实属地管理城市管理执法机制，重点整治交通秩序和环境卫生，提升城市管理水平。运用法治方式推进安全生产和消防安全专项整治，守住安全生产“底线”。全面落实信访条例，主动排查化解社会矛盾，维护群众合法权益。推进律师团挂钩镇（街）、律师挂钩村的“一村（社区）一法律顾问”制度，完成“六五”普法任务。加大打假力度，强化食品药品安全监管，推进“两建”工作，维护市场正常秩序。充实一线执法力量，完善执法程序，落实执法责任制和责任追究制。

深化体制改革。以政府体制改革促进依法行政，推动重点领域改革取得新突破，进一步释放改革红利，激发新常态下的市场活力和社会创造力。深化行政审批制度和商事制度改革，继续取消和下放一批审批事项，完善宽进严管配套措施，扩大政府权力清单编制范围，试点编制监管清单，打造法治化国际化营商环境。深化财政体制、投资体制、科技体制、土地制度和户籍制度改革，激发资本、土地、知识、劳动等要素活力，提高资源集聚能力和配置效率。深化收入分配制度改革、教育综合改革和医药卫生体制改革，建立共享发展成果的体制机制。同时，鼓励基层在农村综合改革、社会治理、新型城镇化、公共资源管理和公共服务提供等方面大胆探索，形成一批可复制、可推广的改革经验。

坚持依法施政。建立政府法律顾问制度，加强政务公开，规范公众参与、专家论证、风险评估、合法性审查、集体讨论决定等行政决策程序，推进政府权力运行的法治化、透明化、科学化。加强法制工作，健全行政复议案件审理机制，纠正违法或不当行政行为。定期向市人大报告和向市政协通报政府工作，重大决策提请市人大常委会审议通过。加强政府系统党风廉政建设和反腐败工作，落实中央“八项规定”、“三严三实”和国务院“约法三章”要求，巩固党的群众路线教育实践活动成果，推行党政机关公务用车制度改革，健全监察、司法、审计等联动的行政权力监督机制，营造风清气正的政务新常态。

各位代表！时代创造机遇，实干成就伟业。让我们紧密团结在以习近平同志为总书记的党中央周围，在省委省政府和市委的坚强领导下，主动作为，攻坚克难，开拓创新，奋力开创揭阳经济社会发展新局面，为实现中华民族伟大复兴的“中国梦”和我省“三个定位、两个率先”目标贡献揭阳力量！
